# What influences the use of HR analytics in Human Resource management in Norwegian municipal health care services?

**DOI:** 10.1186/s12913-024-11610-y

**Published:** 2024-09-27

**Authors:** Kirsti Sarheim Anthun, Kjartan Sarheim Anthun, Erna Håland, Monica Lillefjell

**Affiliations:** 1 Department of Health Research, SINTEF Digital, Trondheim, Norway; 2 Department of Public Health and Nursing, Norwegian University of Science and Technology, Trondheim, Norway; 3 Department of Education and Lifelong Learning, Norwegian University of Science and Technology, Trondheim, Norway; 4 Department of Neuromedicine and Movement Science, Norwegian University of Science and technology, Trondheim, Norway; 5 Department of Public Health, University of Stavanger, Stavanger, Norway

**Keywords:** Human resource analytics, Workforce planning, Structuration theory, Municipal health care services, Norway

## Abstract

**Background:**

The centrality of human resources in the provision of healthcare suggests that Human Resource (HR) management and the use of Human Resource analytics – use of digital data to better understand, assess, plan and organize the workforce - can play an important role in this. However, data driven decision making in the field of human resource management is lagging, and the appropriation of HR analytics in the healthcare sector is limited.

**Aim:**

The current study explores the role of HR departments and the adoption of Human Resource analytics in four municipalities in Norway to obtain insights into what influences the use or lack of use of HR analytics.

**Methods:**

Empirical data were generated through qualitative interviews with fourteen individuals working in HR departments, the municipal administration, and the healthcare services. Structurational theory guided the analysis. The findings show that none of the municipalities made extensive use of data to inform decision making related to human resource management or workforce planning.

**Results and conclusion:**

Three conditions hampered or made irrelevant the use of HR analytics: a decoupling between the services and HR, a weak data-culture, and HR and decision-making processes involving a plurality of stakeholders. However, there were changes underway in all municipalities related to the role of HR and HR analytics.

**Supplementary Information:**

The online version contains supplementary material available at 10.1186/s12913-024-11610-y.

## Introduction

The recruitment, allocation, and retention of healthcare professionals are a major concern among policymakers globally due to present and future shortages of staff [1–4], increased life expectancy, demographic changes, and the rising number of patients with chronic illnesses. In addition, studies have found that insufficient staffing levels in healthcare are not only associated with adverse patient outcomes [5], but staff burnout, decreased job satisfaction and intentions to leave the job have been reported [6, 7].

Staff shortages in the healthcare services have been countered by an intensified search for technological, organizational, and managerial initiatives that can enable the delivery of high-quality services while keeping costs at a sustainable level [8, 9]. As a part of this, attention has also been directed towards facilitating good working conditions in healthcare, and motivating and enabling staff to stay in their jobs [10]. As a result, scholars anticipate that the role and function of Human Resource Management in healthcare will change [11, 12]. It has also been argued that use of various sources of data, including HR data, can contribute to help find solutions [13].

Policymakers have high expectations of the value gains from digital data in healthcare [14], and healthcare organizations globally are currently under pressure to make healthcare more ‘data-driven’ [15]. But so far, investments have primarily been directed to technology that supports clinical work. Administrative infrastructures and functions have received less funding [16]. In line with this OECD reports [17] that countries generally lack systems for monitoring the numbers of health workers.

With growing competition for human resources in healthcare, coupled with more advanced digital systems and access to increasing amounts of data, there is both a need for, and opportunities for, changes of the HR function, and HR analytics (HRA) has been put forward as a key solution [18, 19].

### HR analytics

HR analytics refers to the use and analysis of internal and external data to support decision-making in organizations [12]. Internal and external here refer to data generated within the context of the organisation (internal), or data from outside the organization (external). It has been argued that systematic use of HRA can help predict the need for personnel and turnover, it can assist in competency- and staffing level assessments, improve recruitment processes, and support management and decision making [13, 20]. Making use of data for the good of the employees and the recipients of services has also been emphasized [21].

Based on the growing optimism linked to the benefits of developing more data-driven healthcare services, and the lack of studies investigating barriers and facilitators related to achieving this [12, 22], there is a need for studies exploring the use of HR analytics in the administration, organization, and development of the workforce in healthcare. The present study will contribute to filling this gap in the literature. The aim of this study is to explore what influences the adoption of Human Resource analytics in workforce planning in four Norwegian municipalities’ healthcare services. Our main research question is: what are the factors driving or limiting the use of HR analytics in the four municipalities? Norway displays some characteristics that are relevant in a global context; problem of staff shortages within healthcare and increasing use of digital systems in the administration and delivery of healthcare services.

The paper is organized as follows. We start by presenting research on HR analytics (HRA) before we briefly describe the Norwegian municipal healthcare context. Then follows an outline of the study’s research design and methods, before we move on to report and discuss our findings. And finally, we conclude by suggesting implications for further research.

### Previous research on HR analytics

According to a review of HR analytics literature [23] HRA first appeared in 2004 in the human resource planning literature, where HRA was presented as something other than HR metrics. While HR metrics referred to the measures of key HRM outcomes such as efficiency, effectiveness, and impact [23, p.14], HRA was the use of statistical techniques and experimental approaches that could identify impacts of HR activities [24]. Later, scholars have emphasized the strategic advantages of HRA in identifying the relationship between human resources and organizational performance [23, 25]. HRA should not use only HR data but link a variety of information and data from internal and external sources and perform analytics that should be closely aligned with the organization’s key priorities [23, 25, 26].

The twenty years gone by since the introduction of HRA has seen a gradual increase in research interest, mainly within the areas of organization- and management -studies [23]. Despite this, research has found limited use of data analytics to improve decision-making in organizations [27, 28]. A report [29] showed restricted use of HR analytics among 1200 HR executives from around the world. Only 20% believed analytics would be a primary HR initiative for them in the upcoming two years, and only 12% reported that analytics was a top management concern in their organization.

Research shows that many organizations still lack the necessary skills to conduct HRA [22, 28]. Part of the explanation is that HRA is still in its early stages [23, 30]. Scholars have warned that unless HRA does not shift approach and address the strategic challenges of an organization; hence take HRA “out of HR” ([25], p. 256), HRA will not last but become just another ‘management fad’ [25]. Other more optimistic voices argue that HRA is becoming an established discipline with potential for significant influence on strategic decisions making in organizations [31]. With the fast-pacing developments in machine learning and generative AI, scholars and practitioners also believe that HRA will be significantly improved in the years to come [32].

When it comes to research on factors contributing to successful adoption of HRA, it has been found that senior management’s support is crucial, because managers can provide political support and financial resources for investing in IT infrastructure and software, as well as putting analytics and data-based management on the agenda in the organization [27, 33, 34]. Furthermore, IT infrastructure is also a key factor for carrying out HRA [12, 22, 27, 31], together with the existence of data that makes it possible to conduct meaningful, valid analysis [35]. Related to this, the importance of data exchange and opportunities and potential for integrating data have also been emphasized [36], together with issues such as communication and collaboration between units within the organization, and a supportive culture for HRA [29]. Analytical skills and a data-driven culture are also important preconditions for the adoption and use of HRA [23, 34, 37]. However, one study [38] found that even though the analytical skills of HR professionals such as statistical and presentation techniques are necessary to make use of HRA more effectively, possessing such skills did not greatly influence their adoption of HRA.

Previous research has also reported that HR’s knowledge of the organization, its’ products or services and the key processes of production/service delivery, can improve the validity and quality of the insights from HRA [12, 39]. One study highlights the importance of consultancy skills, visualization, and story-telling skills, as well as translator skills to improve the influence of HRA in the organization [22].

In the growing literature on HRA some have raised concerned voices against HRA and related approaches such as people analytics. They are especially concerned with advancements in machine learning and artificial intelligence and how this will impact HRA and employees (see e.g [40]). The overly optimistic belief in data is seen as problematic as it may lead to mistaking data for reality and installing unrealistic ideas about having control, which again can result in wrong actions. Also, a too strong belief in an allegedly “objective” data-driven decision-making in organizations may result in failure in seeing the need for creative human decision-making, as well as a failure to notice new patterns and opportunities for innovation [40]. The worry is that the use of more advanced algorithms in HRA can lead to reduced autonomy for employees, as it becomes increasingly more difficult to understand the underlying assumptions of algorithms [40]. The problem of reduced autonomy and privacy of employees are also related to the growing amount of data that is generated and gathered, and the potential misuse of such data, for instance in identifying poor performers and introducing a regime of monitoring and control [40]. Scholars therefore call for sophisticated HRA, and a policy of internal transparency to ensure that AI does not become a tool to control employees [40, 41].

### HRA in healthcare

While the research on HRA in business organisations has grown steadily, less research is available on HRA within healthcare organisations [11]. Nonetheless, there are positive expectations within healthcare too on how HR information systems can assist in providing insight that can improve the management, allocation, and retention of staff [13]. Studies report of limited use of HR analytics within healthcare [11], but where it has been adopted, this has depended on the organization’s analytic maturity [13]. A study from Australia found no evidence of sophisticated HR analytics or evidence-based decision-making among line managers in four large hospitals where the official policy was to practice evidence-based decision-making and management [42]. HRA could potentially have improved the line managers’ manual and erratic rostering practice that created a shortage of medical scientists and negatively affected their wellbeing and health. The study demonstrated that HRA was neglected as a tool for adhering to the evidence-based strategy in the organisation.

Another study examined how the use of HR analytics could assist in mitigating incidents of violence against workers in aged care [43]. It was argued that whereas traditional HR has failed to protect staff against such incidents, HR should find new ways to integrate and analyze employee-related data to enhance human capital, improve work experience and work performance among staff. But data was not collected or analyzed on violent incidents and the mental health of staff following such incidents. Investigating what employees needed to cope with such incidents was not done either. Hence, valuable and necessary groundwork in human resource management that could improve the working environment and therefore the retainment of healthcare staff was not done.

### HRA in the Norwegian healthcare context

Increased attention and optimism have been directed towards the use of digital data in the delivery and management of healthcare services in Norway [44], but few studies have explored the use of HRA [45]. We found only one study addressing this issue within the context of municipal healthcare [46]. The mentioned study investigated use and re-use of a variety of data from municipal services, and found that lack of competency, poor data quality, few incentives to use data, rigid laws and regulations, and system retailers’ control of the data, hindered use and re-use of data.

While it has been difficult to find studies in Norway on the use of HR data within municipal healthcare, there are studies looking into the use of digital tools in workforce planning. One study [47] explored the use of digitalized workforce planning in home-based care services and found that this positively influenced the quality and efficiency of the services. The authors did however point out that the tool could have potential positive value for strategic organizational development and suggested this as an important topic for further research. That study, and others [48–50], indicate that while various technological tools and systems are implemented and used in the healthcare services, the effects, outcomes, and experiences from such initiatives are not taken fully advantage of, nor are they being systematically integrated with systems or data in the organization to obtain an overview of the use of resources across units. Studies of what poses challenges for digitalization and data-driven decision-making in municipalities have shown that the most critical hindering factors are lack of ICT competence [51], resistance towards changes [52], and the size of the municipality as this often is linked to lack of maturity in investing in and using ICT systems for the support of services [51].

To sum up, HRA is still at an early stage, and has thus far not contributed to shifting HR’s role from the operational to the more strategic. Research shows that both technological (IT infrastructure, software and digital data) and social issues (various skills, technological acceptance, management’s attitudes, institutional norms, and opportunities for collaboration) influence the adoption and use of HRA. Moreover, the literature identifies a lack of research on HRA in healthcare, despite the very urgent need to explore how HRA can be used in examining how HR initiatives can impact positively on the recruitment, development, work performance and retention of staff. In addition, empirical, qualitative research on HRA within the municipal healthcare sector is very sparse.

The literature also indicates that an exploration of HRA involves much more than implementing a new software tool in the HR department. There is a need for a theoretical approach that is holistic; integrating both the social and material dimensions related to HRA. The present study aims to investigate what influences the adoption of HR analytics in human resource management in healthcare services in four municipalities in Norway.

### The Norwegian municipal healthcare services

Norway has a tax-based national health system where the state and municipalities share responsibility for health care delivery [53]. Hospitals and specialist care services are accountable for health treatment that cannot be performed in community care, while municipalities are in charge of primary care and public health (GPs, maternity care, school health services, long-term care, and social services) [53].

Through reforms and aging-in-place policies, care provision has gradually shifted from specialized hospitalized care to primary health care [53]. This has increased the demands on municipal healthcare services and created greater needs for more advanced medical and health care [54–56]. According to statistics [57] primary health care is the sector struggling the most with recruiting personnel - lacking approximately 14 600 people, and all regions in the country are experiencing more or less the same [57]. Such challenges call for increased attention to how to attract, recruit and retain healthcare personnel.

## Conceptual framework

The theoretical framework informing the analysis is that of structurational theory, as adapted from Giddens [58] by Orlikowski and Robey [59]. Addressing the nature of human actors’ use of computer systems, software, and digital data to inform HR activities in healthcare requires a theoretical framework that facilitates an analysis of the dynamic interactions between subjective human actors and institutional properties, or agency and structure. These make up the duality of social structure [60]. One aspect of this duality refers to how we as human actors can never act or interact in a vacuum, there are always structural properties that influence our interactions. A phrase often heard in organizations is “we do it this way because we have always done so” expresses this notion of adhering to past ways of doing things, confirming those ways but also strengthening their influence and further existence in the present. Social structures thus exhibit properties that are objective in that they provide the conditions for human action to take place. This happens even if we as actors, believe that we act freely, not recognising or acknowledging the structuring properties influencing our actions.

The second aspect of the duality of structure is the human actor, with agency to choose not to keep to the established and the ability to initiate changes in social structure. The duality of structure and agency is always at play, and the relationship between human action and social structure is called the process of structuration [60]. Three modalities are central in the process of structuration. The first modality is interpretive schemes, which refers to standardized, shared repositories of knowledge that human actors use to interpret events and act meaningfully. The second is resources, referring to any means used by human actors to reach their goals, and exercise power (money, tools, skills, knowledge). For instance, in healthcare organizations clinical professionals will often have a high standing, and their professional status can be drawn on as a resource that can influence social situations. The third and last modality is that of norms, which refers to the rules for proper conduct.

A structurational framework allows insight into how the social structures of an organisation are influenced by the use of informational technology and how the use of informational technology is influenced by the social structures of the organisation. Examining only one side of this will provide a partial understanding of how organizations and technology, interact. In the structurational framework technology does not determine social practices. There is always the possibility that organisational members may choose not to use technology in the way it was supposed to be used, or they may choose not to use it at all. Human actors’ values, interests, knowledge, skills and professional norms and power influence how they interact with technologies. They will invoke such social structures to either undermine the use of new technology or they will transform social structures in the organization through appropriating the technology.

In the present study the structurational model of technology will provide a lens through which we examine the interactive relation between humans, technology, and organization. Our aim is that attention to this, can unravel the complexities inherent in the use of technological artefacts in the management of municipal healthcare services.

## Methods

This qualitative, interview-based study was conducted in four medium-sized- to small municipalities, focusing mainly on the HR department in each municipality, and addressing the HR departments’ work in relation to the healthcare services, and especially the use of data and analytical-work within their practice. Research-data regarding experiences with the role and function of HR and the use of HR data and other sets of data were collected. All interviews except one were individual semi-structured interviews, which allowed participants time to share and elaborate on their experiences, thoughts, and opinions without the interference of other interviewees. The one group interview was conducted with two persons from the HR department in one municipality: the Senior HR executives and a HR consultant. The reason behind conducting a group interview was that the Senior HR executive had just started in the position, and therefore wanted someone present that knew the HR departments’ role and work more thoroughly. All data collection was carried out by the first and second author, except two interviews that were conducted by the first author only.

### Selection of participants

The municipalities had been purposefully sampled to ensure variation in size and characteristics pertaining to the rural-urban divide. Interviewees were identified and selected purposively and sequentially [61] by the research group. First, we recruited individuals working in HR departments – such as HR consultants or HR executives – as they were thought to possess pertinent experience and knowledge directly related to the issue studied. One of the municipalities had only one HR executive and no HR consultants, and one HR consultant had to turn down the invitation to participate in the interview due to workload and time-pressure, thus only two HR consultants were interviewed, and four HR executives (one HR executive from each of the municipalities). HR executives and HR consultants in all four municipalities stated that HR work was split between the HR department, the head of municipal affairs and section leaders from the healthcare services. Therefore, we decided to recruit interviewees from the senior management level and among section leaders in the healthcare services, making sure that we had at least one of each of these categories from all four municipalities. Inclusion criteria were responsibilities in human resource planning and human resource management related to the healthcare services. Consequently, we interviewed in total fourteen individuals - four HR executives, two HR consultants, four Heads of Municipal Affairs, and four section leaders from the healthcare services. Out of the fourteen interviewees, ten were women. Two HR consultants and two heads of municipal affairs were interviewed a second time, allowing us to probe for more information on their use of data in HR related tasks. Participants were contacted by e-mail and received both oral and written information of the study and about their rights to withdraw from it at any time without explanation. No one dropped out during the study.

### Data collection

Interviews took place during the last two quarters of 2022 and the first quarter of 2023 and were conducted mainly in a conference-room in the municipality council hall, except three that were conducted digitally. All participants had worked in their present role for 1 ½ years or more, except for one HR executive who had started just four months prior to the interview. Interviewees were provided information about the aim and objectives of the study and that participation was voluntary. All participants gave their written consent. Interviews lasted for approximately one hour and were digitally recorded in their entirety, and all except two were transcribed verbatim. Careful notes were taken in the two interviews that were not transcribed verbatim. The interviews were conducted, transcribed, and analysed in Norwegian. Only the quotes used in this article were translated into English. The analysis was performed on the original interview transcripts. To ensure correctness of the translation, the quotes were double-checked by the members of the research team, who are proficient English speakers.

The interview guide was developed and shared in the researcher group. It featured open-ended questions that could bring out participants’ thoughts and reflections. The interviews were explorative, and the interview guide included questions about work-role, tasks and how long interviewees had worked in their current role, and they were inquired about their thoughts on human resource management, their use of ICT systems and the software applications, and the use of data as a basis for decision-making in matters related to human resource management. The interview guide can be viewed under the Supplementary files. All interviews were conducted on the basis of respondent and organizational anonymity. Interviewees would only be identified by the role title,

Based on the classification scheme of Statistics Norway [62], one municipality (M1) can be characterized as a medium sized, urban municipality. The second one (M2) is a medium sized sub- or peri-urban municipality, while the last two (M3 and M4) are rural municipalities, one medium sized and one small^1^. See Table 1.

**Table 1:** List and brief information on the municipalities included

Name of municipality	Size	HR: man-labour years^a^
M1	Large sized, urban	6
M2	Medium-sized peri-urban	4
M3	Medium-sized rural	1
M4	Small-sized rural	4

^a^These resources work for all sectors in the municipality, not just for the healthcare sector.

### Data analysis

To examine the data, the study followed the principles of thematic coding [63, 64], allowing us to identify, analyse, and interpret patterns of meaning, or themes, in text-based data. Analysis was performed in conjunction with the data collection process, permitting any indistinct questions to be probed further in subsequent interviews. The first author read and re-read the material to obtain an overview and impression of the material. Features interpreted to be important in answering the research question were coded into initial codes. The codes were discussed among two of the researchers who had conducted the interviews, and potential themes were decided on. All the coded data relevant to the themes were then collated. Codes and themes were then discussed in the whole research group. Themes were then checked in relation to both coded extracts and by returning to the interview transcripts. The themes were discussed again in the researcher group, to develop themes that reflected the ‘essence’ of the material while also responding to the study’s research question and to make sure themes held true across the four municipalities. All the themes were then considered in terms of how they related to each other, and the theoretical framework chosen for the study. We revisited the list of themes in a critically reflexive and evaluative way until agreement was reached. Credibility of the analysis was enhanced not only through researcher-triangulation, but also through conducting a member check [65]. The study results and quotes were presented at a workshop with managers and some section leaders from the participating municipalities for feedback on the findings. Findings were found to reflect the situation in HR management in the municipalities.

## Results

The aim of our study was to obtain insight into what influences the adoption of HRA in human resource management in the healthcare services in four municipalities in Norway. The structurational perspective [58] informed our study, especially its application in studies of information systems [59] since the literature indicates that the appropriation of HRA involves an emergent interplay of structures and agency in organizations. Analysing the interview transcripts, we found that none of the municipalities made extensive use of data from HR to inform strategic human resource management. The organizational context encompassing the use of, or rather, in this case, the lack of use of HR data and HR analytics, was characterized by some structural conditions that contributed to an undermining of HRs ability to take on a more strategic function in the organization. Adopting a more strategic function has been highlighted as key in developing a strong HRA culture [25, 66], as this improves HR’s ability to perform HRA that are strategically important and thus will have impact.

We have organized the findings in four themes. The first theme we have called a decoupling between health services and HR in matters related to HR management. This included statements and reflections from interviewees describing a divide between the healthcare services and HR where HR performed the administrative tasks related to human resources, while the section leaders did the practical day-to-day human resource management such as staff rostering, competency planning, and skills development. A second theme common across municipalities was weak data-culture with a lack of resources in terms of proper IT systems and knowledge and skills to carry out HR analytics. The third theme had to do with the need to satisfy a plurality of stakeholders rendering strategic and long-term recruitment planning difficult. In sum, these social and technological structures have led to weak emphasis on and adoption of HRA. However, as a fourth theme, the analysis revealed that certain changes are under way and that some actors, especially within the HR departments, saw a need for change. In the following the themes are presented in more depth.

### Decoupling between the health services and HR in matters related to HRM

Employees in the HR departments across the four municipalities described their work in a similar fashion where a large share of the work is dedicated to administrative work related to payroll, recruitment and hiring, employee benefits and sick leave following-up. HR was also involved in committee-work and was responsible for facilitating the collaboration between trade unions and the employer. HR was brought in in an advisory capacity in relation to rules and regulations, and in a practical capacity, handling formalities in the hiring process. One of the HR executives described HR’s work as follows:

HR does recruitment and development of the HR unit. We are involved in organizational development, but at an overall or systemic level. We are responsible for following up on matters related to health-environment- and safety-regulations, and processes linked to salary negotiations. Each of the section leaders are responsible for the recruitment processes in their sections, but we support them when they hire new staff. And HR deals with reallocation of staff within the organization (HR executive, M1).

All HR departments are organized directly under the chief municipal executive, with the HR executive serving in the municipal executive’s cabinet. The HR departments comprise 1–6 employees, including the HR executive. In three of the four municipalities the HR consultants serve all municipal sectors, meaning none of the HR consultants are dedicated to one sector only. One of the HR consultants even said, “You have to be a generalist working in HR”. In the fourth municipality, the HR consultants had recently divided the sector areas between themselves on a permanent basis. This change was initiated when an employee transferred from one of the sector areas to the HR unit. Then it seemed reasonable to dedicate that person to the sector area s/he knew well, and the remaining HR consultants were equally assigned to a specific sector area. Since this was a recent change, they had not registered specific results related to this, though the HR executive said this could be the case in the longer run.

The HR departments were in the view of the section leaders expected to take on a support role for the municipal executive and the different administrative sectors such as health and welfare, education, and technical services. While section leaders acknowledged and appreciated HR’s competency on laws and regulations in matters related to the administration of personnel, they would underline the necessity of first-hand knowledge about the ward, the patients, the clinical work and the staff to make the right decisions in the day-to-day organization of human resources. HR was too far removed from clinical work, thus could not be involved in making decisions about staffing or competency planning. Section leaders pointed to how the services must deal with an increased demand for specialization in healthcare, as patients have more complex and serious conditions. “We’ve become a mini hospital”, said one of the leaders at a nursing home (M3), and added that each ward at the nursing home faced unique challenges based on the skills-mix that the staff represented and the group of patients they care for. This development strengthened the need for more detailed knowledge about the services when organizing resources and competencies in the services. The changes within healthcare were also recognized among the HR professionals in all four municipalities. One of the HR Executives saw that a closer collaboration between HR and the services could be required in the future:

There are competency plans for the healthcare services, but you’ve got to talk to the section leaders about that. The municipal healthcare services now and ten years ago, that is not the same when it comes to the advanced healthcare service that is being provided today. But in HR we are not involved in that. But perhaps that is something that HR *should* be involved in in the future? (HR Executive, M1).

Interviewees from the services in all four municipalities were however content with the current relationship and division of work between services and HR, and in two of the municipalities (M2 and M3), the HR consultants were also content with how things were. They said that HR had enough to deal with as it was. One of them added that HR wasn’t supposed to do much else besides operational and administrative tasks linked to hiring and following up on those who were on sickness-leave. Implicit in this there was an understanding that HR professionals should keep to their work as bureaucrats.

As a result of this, human resource management had to be divided and be part of management at all levels of the organization. Resource planning, staff rostering, and competency planning- and management were taken care of by the section leaders, whereas the more overarching issues related to management of resources were discussed between section leaders and the Head of Municipal Affairs. Personnel administration was performed by HR. One of the Heads of Municipal Affairs stated in relation to this: “HR-work is probably done every other place except where the HR-competency is located”. The result, according to the same person, was that the more strategic side of human resource management could suffer, since no one would be able to grasp the whole picture and think and plan on a longer-term basis. The interviewee also claimed this was part of tradition and how things had always been, thus difficult to change.

In sum then, HR is mainly involved in routine administrative work, *servicing* the healthcare units, ensuring that recruitment and exit processes are dealt with according to laws and regulations. Historical and cultural expectations and norms about who should do what and who serves whom result in a decoupling between HR and the services. Descriptions of their work and professional priorities indicated that they adhered to different institutional logics; that of public administration (HR), and that of professional clinical work (section leaders). The different institutional logics were maintained in their interaction and by the institutional set up and routines, resulting in a continued decoupling and what can be termed a siloed or compartmentalized structure when it comes to HRM work.

### Weak data-culture

In all four municipalities HR collects, registers and monitors data, meaning; HR does a fair share of ‘data-work’ [67]. However, as we will see, this is not systematically curated to be used as input for workforce planning, human resource management or to inform strategic decision making. Common to all four municipalities was a weak culture or weak social attribution to data-driven or evidence-based decision making.

An organization’s data-culture refers to organization-wide structures, practices and capabilities related to the production, collection and utilization of data [68, 69]. Thus, it is not limited to the IT department’s capabilities and resources, but includes all organization members’ capabilities to find, analyze and utilize data, as well as the institutional structures necessary to support this [68, 69]. This is why we in this theme include statements related to data awareness and skills, work and routines in performing data-work, and material/technological structures supporting data-work, such as computer systems and software.

The data-work that HR does is mainly linked to statutory reporting and regular reporting to national authorities and registries. The reporting included data on absenteeism, numbers on full-time equivalence, and employees’ working hours. HR also reported internally to the municipal administration and to the municipal executive board on many of the same issues. HR was also responsible for conducting work environment surveys every second year and attending to policies and follow up of pay and benefits. All of this would equip them with some data on the workforce. They would however complain that different sets of data were registered in different systems, making it cumbersome to integrate data or perform analysis across the data.

Interviewees from HR expressed a wish for more accurate and digitalized workforce data. They also said that moving from several different software systems to a more advanced and integrated system would make life easier and contribute to more efficient workforce planning and recruitment processes. One of the HR executives pointed out that they could all (Heads of Services, section leaders, Heads of Municipal Affairs) sit down and discuss the recruitment situation, but without any data, this was somewhat pointless. The HR executive also said that HR *could* make an effort, curate data, and present a more accurate basis for decision-making, but it was tedious work given the state of data and the fragmented nature of it. Interviewees from the HR departments also said they still use simple spreadsheet programs for data analysis (i.e., Excel).

All interviewees described a similar situation where they worked in several different software applications that all performed different tasks (i.e., one for payroll and benefits, one for recruitment, and one for archiving information on employees such as hiring date, position, education etc.).

Most interviewees from the top-management level and the healthcare services in the municipalities did not see the value of data and HR analytics in supporting decision making in human resource management and issues related to staffing. Strategic planning by use of historical data or statistics was not something they saw as necessary or relevant. At the same time, however, they expressed worry when it came to how healthcare could recruit and retain human resources both in the near and far future, and several stated that something had to be done, but HRA was not mentioned as a potential solution here. Section leaders made decisions founded on concrete and first-hand insight into patients, staff and work-practices, without seeking support from data or initiating efforts to systematically assess dynamics between such factors over time. One of the section leaders complained that an increasing amount of computer work has been introduced into healthcare and stated:

All of them [the computer systems] are a little bit different, and we must learn these systems. My staff complains and says, ‘This is not why we started working in healthcare – working with computer-systems instead of people’ (section leader, M3).

One of the HR executives uttered matter-of-factly that:

I don’t believe there is a tradition to think strategically about HR in the public sector. We use HR statistics and data too little (HR executive, M1).

The section leaders interviewed did not see how HR could contribute with analytics work that could be valuable in the strategic planning of human resources. Nor did they see HR data as relevant or useful for the management of the services. “How could we use such data?” one of them asked. Another unit manager uttered: “I wouldn’t know what to ask for. What data would I need?” (section leader, M2). Both statements indicate a lack of awareness and skills in interpreting and using data.

Both at the operational (service level) and strategic planning levels (among chief municipal officers/leaders), there seemed to be a lack of attention to promoting work practices supported by HR analytics, nor did there seem to be a widespread recognition of what could be gained from HR analytics. For instance, in one of the municipalities (M3) various measures had been implemented in the healthcare services, but no systematic assessment of the outcomes of the measures had been performed. The measures involved flexible roster arrangements, options for staff training and development, and job crafting opportunities for employees who in periods experience reduced work capacities. Having HR follow up and assess for instance employee work satisfaction or absenteeism prior to and after implementing such measures could have given important insight into what influences sickness-absence and work engagement positively and negatively.

Spreadsheets were used in HR and among section leaders in all four municipalities, but data around work intensification were not collated to determine human resource needs, nor were data on the human resource situation and related issues such as absenteeism, turnover rates, patient outcomes, leadership span or social climate being systematically curated and analyzed to learn more about staff requirement.

When queried about whether managers at different levels asked for HR analytics reports, HR professionals said “no”. One of them explained that this was also due to poor data because of outdated computer systems:

They do not ask for such reports […] We have some data. And they can ask us for some reports, but we do not have much data really. And the data we have is a little bit inaccessible because our system is somewhat outdated (HR consultant, M2).

In none of the municipalities did the services use HR data or HR analytics as a basis for decision making or planning. Interviewees said that planning and allocation of human resources were done without the support of data from HR or HR analytics because the size and scale of it didn’t require it and because section leaders did not have time to do so.

They [section leaders] don’t use [computer] systems for that. This is rather something they […] have in their heads. They calculate such things like the duration of a duty, the duration of lunch breaks, other breaks, and they know their employees. […] And the reason for it not being used much is related to time-pressure. When you are pressed for time, you go for the most critical tasks, those that are rather short term, …you take what is ‘burning’, and that is staff rostering […]. But the bigger picture, with numbers and stuff like that … no one takes time to go into that (HR consultant, M4).

When asked whether HR performed any predictive analysis based on historical data from the services, the interviewees said no. Nor did HR seem to conduct any prescriptive analytics that could be used as support or basis for decision-making by the section leaders when doing workforce planning. The section leaders did not require reports or statistics that could be organized, analyzed, or translated for the purpose of planning at service level. In general, little seemed to be requested from leaders at the various levels in the organizational hierarchy. One interviewee from HR posited that HR could produce some reports, but that they didn’t, since it wasn’t requested from them:

Municipal leaders and politicians ask for reports on the share of employees working full-time. Other reports are not asked for. We can create various reports, but these are not asked for upwards in the organization. They request very little. […] We *can* produce some reports from our current HR system, but we don’t use this much (HR consultant, M2).

During interviews HR consultants and HR executives in all four municipalities were very open about the problem of a general lack of data. This became especially apparent when it came to mapping, or the failure to map, competency profiles in the healthcare services. They had compiled information about employees’ job title and level of education but lacked details about employees’ skills and exact competencies. Employees could register this type of information in the HR system themselves, but few had done so. This recurring statement on the lack of good data on the staff’s competency can be exemplified by two HR consultants, who said:

I think the challenge is about not having a proper overview of the competency available among staff. We don’t have a system, or we have a system, but the system is outdated when it comes to registration, and too few of our employees have registered any information in the competency module. […] The codes for competencies are outdated and do not reflect for instance that an employee has a master’s degree in such and such a discipline (HR consultant, M2).

We have an HR system where employees can register their competency, but not everyone has done that. Every unit should have an overview of the competency available at the unit, but this is currently not the case (HR executive, M4).

Outdated computer and software systems were also a problem in all the municipalities. Working in several separate systems was time-consuming since information often had to be registered numerous times in the different systems and it made it hard to obtain a good overview of the workforce through workforce data. One of the HR consultants said this also contributed to a distrust in the quality of data, as one could not be sure whether people remembered to manually double and triple register information.

The interviews also revealed that even though HR professionals saw the need for more data-driven decision making when it came to human resources management, the computer systems they had to work with, and the data they had available created difficulties when trying to do analytics work. One of the HR executives (M1) found this frustrating and said:

And recruitment, what are we dealing with there? We may have opinions about several issues, but without having the facts and knowledge straight….? […] We lack data to support our decision making. We sometimes try to develop reports on these matters, but those are generated manually. HR has done that, but it is very cumbersome.

Overall, this theme shows that the data-culture in the municipal organization around HR and the healthcare services is weak. There is a lack of awareness of how data can inform decision making, and a lack of proper IT software to collect, curate, analyze, store and monitor data. Additionally, the data that is collected is stored and handled in separate systems. The state of data is also said to be quite poor or inconsistent.

### Plurality of stakeholders

When asked to reflect around why HR was not involved more in the strategic management of resources, the interviewees would point to the highly institutionalized nature of the healthcare sector and how laws, regulations and bureaucratic processes contributed to rigidity, which again made strategic human resource management difficult. Tedious democratic processes left little room for HR to make any real calls, or would drag out decision making processes to the point where decisions needn’t be made:

Things take longer time here, compared with the private or business sector where there is more leeway to move things forward more quickly. We must work very hard sometimes to get approval from everyone involved. And sometimes when we finally get those green lights, the case has been closed. There are a lot of red lights here (HR executives, M4).

The need to involve a wide selection of stakeholders also contributed to the slowness and the rigidity:

You need to have the different groups on board if you want to get things done here. A lot must go through the trade unions. There’s loads of involvement, approvals, discussions, and negotiations before you can get any real ‘action’ (HR executive, H4).

One HR consultant exemplified the rigidity in the sector by discussing the hiring process when there is a vacant position:

For instance, when a nurse leaves his or her position, this nurse’s position is advertised as vacant. It must be discussed and negotiated with the trade union if the competency requirement for that position is to be altered. So, we [in HR] must keep firm control over this (HR consultant, M4).

One HR executive stated that they [the municipality, and the healthcare services] were falling behind and not really *managing* matters in the way they should when it came to staffing. Certain laws would set limitations. For instance, part-time workers who during the past twelve months had regularly worked more than agreed working hours were by law entitled to a post equivalent to the actual working hours during this period. The HR executive said that the municipality often saw that small and temporary positions would fail to attract the necessary competency to the services, but following the law, they were forced to let employees with a lack of competency or a different competency than what was needed work their way into larger and permanent positions. The services would then still lack nurses or social educators. The HR Executive saw this as a failure to perform strategic HRM.

Sometimes I think we tend to practice strategic ‘poor governance’, unconsciously. […] During the last year we have handled many such claims on extended positions. Most of these claims are redeemed, but by redeeming these claims, we lose our ability to manage competency strategically (HR executive, M3).

Some HR professionals believed HR should be consulted more in such matters and that HR could contribute to finding better, long-term solutions together with all relevant stakeholders, thus it was an issue that had to be discussed at the regular meetings with the trade unions and the managers in the municipal organisation. One of the HR consultants did however express worry that since many stakeholders were involved, such as trade unions, managers from different sectors and services, and HR, this could make things difficult. Stakeholders would typically focus on their key interests or concerns and would fail to see the whole picture. According to this person, there was a need to address the manner of collaboration too:

I think that we should talk more about how we can improve our collaboration to develop the health care services. […] We should gather all stakeholders and develop a common understanding of the goals and how we can contribute to deliver good services while aiming to innovate at the same time (HR consultant, M2).

Politicians in all four municipalities had put pressure on HR and the services to increase the share of full-time employees, ordering an investigation of involuntary part-time work in one municipality and demanding regular reports with updated numbers on the full-time equivalent in two of the other municipalities. The HR professionals were divided on how positively they viewed this political pressure. Some would for instance question the politicians’ interest in mere numbers, without looking into the causes behind the high share of part-time work. In addition, some expressed worry about political hot topics, political symbol-making and opposing signals from the national and local political levels. This is exemplified in the quote below.

Back in 2020, there was a political decision in this municipality to report on and investigate how many employees were working involuntary part-time. That was a political order. We [in HR] started working with that quickly, thinking that it could be useful knowledge. But we wanted to look at the causes here too. What drives part-time work? The general idea around here was that employees in the healthcare services cannot work full-time because the work is too hard. So, is part-time work something that is involuntary or is a choice that the employee makes? We found that it was a combination. And at the national policy level there is this idea that full-time should be the rule in healthcare, hence we should not investigate whether part-time is voluntary or involuntary since that sends the wrong signal and opens for part-time at the wrong terms. But in healthcare you need to have staff twenty-four-seven, creating some definite challenges when it comes to working hours if everyone works full-time, thus we can’t do without part-time employees (HR executive, M1).

HR professionals would also highlight the need to see different concerns in combination and several expressed concern that the different stakeholders would often fail to do so. Trade unions had their interests, politicians theirs, and managers at different levels and in different sections would be concerned with their specific issues.

This third theme highlights how public sector HR practices are characterised by a high degree of institutionalization; as they are influenced by laws, regulations and influence from politicians at different levels and trade unions. As a result, the strategic management of human resources becomes difficult. The theme also shows that the adoption of HR practices is embedded in specific organizational structures and cannot be seen separated from those.

### Changes are under way

In all four municipalities, the implementation of more advanced HR systems was either on the verge of taking place or were to be implemented in the upcoming year or two. One of the HR consultants said the municipality had signed a deal with the contractor for a new cloud-based system that would provide HR with far better tools to work with, a system where data could be stored, integrated and searched for more easily. A substantial digital upgrade was on its way in another municipality, but the financial situation hindered that the upgrade could be done all in one go. It had to be portioned out over a few years. In two of the other municipalities, HR professionals had high hopes for the HR Information System that was soon to be installed:

We have an HR Information System. We added another component to it not so long ago. Now we can use it as a system that can support management and that can provide us with an overview of employment positions. This is real-time data on everything related to personnel, sickness absence, full-time positions, part-time positions. Nearly down to the level of individuals. It can also provide information on how much we spend on temporary staff and stand-ins, and how much we spend on sickness absence. By acquiring this system, we want to give the managers an easier life, provide them with HR information that can support them in their work as managers (HR consultant, M2).

Soon, we will implement several new digital systems, and the aim is, seen from the management level at least, that we should make better use of such systems to develop the services and move forward (HR consultant, M4).

With the new system that will be implemented, we can start mapping the competency of our staff. And it will be easier for the managers of the services to develop competency plans for the service and for the individual employee, according to what is needed in the future (HR executive, M4).

Interviewees from HR departments in all municipalities saw that HR could play a more significant role in strategic human resource management compared to what was the situation today. Workforce shortages in the healthcare services had brought the issue of human resource management to the forefront, both locally, nationally and globally. Locally, one HR executive alluded to how a variety of the municipality’s concerns converged in the issue of resource use. Subsequently, by lifting the issue out of separate service units, collaborating at a higher organizational level, solutions could be found, and HR, who served the whole organization could play an important role. However, the HR executive also saw that there was a long way to go before getting there:

Using our resources effectively and wisely is touching upon a range of issues; it is about focusing on increasing the full-time equivalent, sharing the nursing resources, collaborating more extensively across units in healthcare, and strategically *managing* the competency in the services. We have a long way to go there in municipality healthcare services (HR executive, M1).

The same HR executive added that since human resources were in shortage, the solutions to the future delivery of services could mainly be found in the use of these resources, something that should make HR more central:

There is a shortage of competencies in the healthcare services. At the same time, we are required to deliver the same secure and sound services and to follow the laws and regulations. But people cannot just run faster, that is only possible to a certain limit. Instead, we must use our resources more cleverly. HR should play a role in this (HR executive, M1).

One of the other HR professionals believed s/he saw signs of a growing awareness of the need for data-driven decision-making in human resource management:

I think our municipality has a job to do here [in the strategic management of competency]. What do we need now? And what about the future? [...] I think the management is quite aware that we need to improve when it comes to HR and using our computer systems in a way that contributes to developing the services (HR consultant, M2).

## Discussion

What has been explored in this study relates to the increasing crisis of staff shortage within healthcare systems globally. At the outset, we referred to the demographic changes that are posing challenges to the Norwegian municipal healthcare services. We posited that this would bring forth some changes in strategies of municipal administrations, and that especially HR would come to see the use and analysis of HR data as valuable input to strategic management planning. We wanted to investigate if this was the case in the four municipalities participating in this study.

We made use of structurational theory [58] that could unravel the dynamics between social and technological structures and the interactions between the two and enable an understanding of how this relationship influenced HR work. This framework allows insight into the structural properties that enable human actors to stick to or deviate from old structures. The data showed that social structures; pertaining to the institutional context and the human actors (interpretive schemes, resources and norms) constrained the use of such information technology, data, and therefore HR analytics work, HR data or HR analytics was not asked for in the organization. Lack of insight into how it could be used, motivation and skills to use it were mentioned as barriers here. Also, it seemed that forms of reasoning or systems of rules, professional ethics, laws and regulations, drove decision-making in recruitment and staffing. These social structures worked as disincentives to use HR data in such matters.

Moreover, human actors were engaged in different social realities that put different weight on the added value of HR data. Section leaders did for instance emphasize getting the short-term workforce planning in place, and there was a tension between getting the staff planning ready and finding time to go into a more thorough and long-term strategic planning. Digging deeper into, or pursuing, the long-term strategic resource and competency planning would involve time and efforts that they appeared not to have. They seemed uncertain about the value of HR beyond the support function HR played in relation to the services. Several HR executives and consultants did see some advantages of using HR data for strategic planning and resource allocation, though found it too difficult to overcome the norms, values, policies and practices in other contexts (healthcare services, trade unions). Also, they saw it as the top management’s call to acknowledge the potential of HR analytics and invest in the proper material artefact that could realize this potential.

The technology’s or software system’s structural properties constrained the human actors in especially two senses: one was pertaining to interconnectivity; the other was that of data-quality. When it came to interconnectivity, the different software applications were not connected or could not communicate with each other, preventing the transferring of data from one to the other, or the cross-utilization of data. Interviewees said this made the use of data less valuable, and efforts to make data more usable involved too much work. Poor data quality also contributed to making HR data less valuable, less usable and less reliable. Financial resources are tight in municipal healthcare, and thus far such technological systems have not been prioritized. This may also be a result of the stronger power and influence of certain professional groups over others. As pointed out by Bach [69] HR must transcend its own functional boundaries and be more involved with the processes in the services, in the planning, execution and evaluation of important initiatives. If not, the HR function in the health sector must remain in a marginal position. According to Bach [69] HR’s legitimacy and worth require that HR can show how it contributes to improved patient care. The status and standing of healthcare workers as public servants and professionals are also significant here. By tradition these services are associated with regulation of work conditions and high levels of job security. This can undermine the potential for HR to adopt a more strategic orientation. Bach and Della Rocca [70] have argued that the status of healthcare workers as public servants prevents the disruption of health services, and this is a way of ensuring standard and professional conduct. But through closer collaboration between HR and the services, HR can address the most critical challenges experienced in the services, and thus generate input that contributes with a focus on employee-centered outcomes that are linked to the performance of the services.

Furthermore, Dremel et al., [71] have argued that digital transformation demands a data-sharing and data-driven culture where data is recognized as a valuable resource and enabler. Hence, attention should not only be directed to the role of digital technologies, but also to the dynamism between organizational units and how this influences the investments in technologies and which organizational processes and tasks are supported by digital technologies are important [72]. Changes that need to be considered do not only involve the individual users’ ability to handle the technology and learn how to adapt to it or how to “lift” one’s work into the technical realm. Matters are more complex and may involve another way of thinking about work and what HR can do. As Kraus et al. [73], has aptly remarked, there is a need to build more holistic adoption models for the implementation and use of information technologies within healthcare structures [73].

Our findings are in line with research done elsewhere. The HR function in healthcare plays a marginal role and is underdeveloped, and leaders, clinicians and support staff fail to understand the value of analytics as a tool for decision making [74–77]; data quality is poor [78, 79]; the HR function may lack the skills, knowledge and insights to ask the right questions of the HR data they have access to [25], HR is not posited sufficiently at the core of the services’ strategy work [25], HR and the services are loosely coupled and do not join forces to develop sound strategic policies [77, 80, 81].

In a report assessing the healthcare services in Norway [81] it was concluded that despite the critical role of planning and analysis for tackling future demand for healthcare services, most municipalities have been forced to shrink their administration, and thus have limited capacity and competency in analysis and planning of services, including providing needs analysis and service-profiling [81], p. 31). The Office of the Auditor General of Norway has also posited that the state lacks a proper overview of the municipalities’ efforts in ensuring right capacity in eldercare and that little work has been done locally when it comes to analysis and planning [82].

### Methodological limitations

Some cautions should be taken in interpreting and extrapolating the results from this study, since this is a small scale, qualitative study conducted within the context of one country. However, the findings are in line with studies conducted elsewhere, i.e [12, 25, 74]. There is a need for further research on how improved data on the workforce in healthcare, in combination with data on service delivery, can contribute to improve workforce organization and planning, as well as how it can be used to support the recruitment and retainment of employees. More research on how better use of workforce data and planning can impact on patient care and healthcare quality is also needed.

## Conclusion

Our study found that the adoption of HR data and HR analytics were restricted by a combination of social rigidities such as responsibility allocation, structures for interaction and collaboration, hierarchies and detailed policies guiding recruitment and hiring, and poor software systems and data. What is studied here suggests that the municipal healthcare system has not yet been significantly transformed technologically when it comes to HR data and HR analytics. The management of healthcare services is not data-driven to a great extent, but a change is gradually taking place and will perhaps be inevitable against a background of wider changes associated with expanding digital data-systems where social spheres are mediated, linked, and monitored digitally to an increasing extent.

A note of caution should be made, however. Care work is a form of work that has its own logic [83] and it is an ongoing process between the care-provider and the care-receiver that to some extent is unpredictable and therefore resists standardization [11]. Thus, we agree with Francis & Keegan [21] who argue that the HR profession needs to reflect on the framing of HR work, and how HR should contribute with a focus on employee-centered outcomes that are linked to the performance of the services. Analytics work can be dedicated to predicting resource needs, identifying what leads to employee job satisfaction, and exploring ways of freeing up resources. Good working conditions are a critical precondition for attracting and retaining human resources to the services and for providing quality services.

This study’s findings have implications for research and practice. As socio-material entities and the dynamism between them influence technological transformation, research should exercise caution and not mistake the social as only related to human skills at the individual or microlevel but acknowledge that technological transformation is also significantly related to the larger institutional context. HR practices and use of HRA needs to be contextualized when studying organisations.

For practice, this study’s findings imply that rigidity or slowness is an integral part of technology transformation. There is a need for a balanced view on transformation. National governments might well emphasize the urgent need for change, without much understanding of the need to invest sufficiently in enabling social entities such as different institutional contexts *within* a larger organization to cope with increasing tensions resulting from differences in the pace of how easily work practices and mental models of work can change, and new skills be learned.

## Supplementary Information

Supplementary Material 1.

## Data Availability

The primary data of this study consists of qualitative interview transcripts, which were exclusively carried out for the purpose of this study and have not been published elsewhere. To protect interviewee confidentiality they are not publicly available. Request regarding the data should be made to the corresponding author.

## Footnotes

1 According to Statistics Norway’s classification scheme [62], municipalities with 20 000 or more inhabitants are large sized, municipalities with 10 000 to 19 999 inhabitants are medium sized, whereas municipalities with less than 10 000 inhabitants are small sized. 81% of the inhabitants in Norway live in the hundred largest municipalities, with more than 11 000 inhabitants. The average number of inhabitants in Norwegian municipalities is 15 000.

## References

[1] de Vries N, Boone A, Godderis L, Bouman J, Szemik S, Matranga D, de Winter P. The race to retain healthcare workers: a systematic review on factors that impact retention of nurses and physicians in hospitals. INQUIRY: J Health Care Org, Prov, Financing 2023:60. 10.1177/00469580231159318 PMC10014988 36912131

[2] Kroezen M, Dussault G, Craveiro I, et al. Recruitment and retention of health professionals across Europe: a literature review and multiple case study research. Health Policies. 2015;119:1517–28. 10.1016/j.healthpol.2015.08.003 26324418

[3] Dahl HM, Hansen LL. Chapter 1: introduction: a care crisis in the nordic welfare states? In: Hansen LL, Dahl HM, Horn L, L, editors. A care crisis in the nordic welfare states – care work, gender equality and welfare state sustainability. Bristol: Bristol University; 2022. pp. 1–15.

[4] World Health Organization. World health statistics 2022: monitoring health for the SDGs, sustainable development goals. Geneva: World Health Organization; 2022.

[5] Ball JE, Brunyeel L, Aiken LH, Sermeus W, Sloane DM, Rafferty AM, Lindquist R, Tishelman C, Griffiths P, Consortium RNC. Post-operative mortality, missed care and nurse staffing in nine countries: a cross-sectional study. Int J Nurs Stud. 2018;78:10–5. 28844649 10.1016/j.ijnurstu.2017.08.004 PMC5826775

[6] McHugh M, Ma C. Wage, work environment, and staffing: effects on nurse outcomes. Policy Politics Nurs Pract. 2014;15(3–4):72–80. 10.1177/1527154414546868 PMC4667784 25121923

[7] Drange I, Vabø M. A cross-sectional study of sustainable employment in nordic eldercare. Nordic J Working Life Stud. 2021;57:103–24.

[8] Vabø M, Zechner M, Stranz A, Graff L, Sigurðardóttir SH. Is nordic elder care facing a (new) collaborative turn? Social Policy Adm. 2022;56:4.

[9] Hoppania H, Olakivi A, Zechner M, Näre L. Managerialism as a failing response to the care crisis. In: Hansen LL, Dahl HM, Horn L, editors. A care crisis in the nordic welfare states – care work, gender equality and welfare state sustainability. Bristol: Bristol University; 2022. pp. 109–30.

[10] Kabene SM, Orchard C, Howard JM, et al. The importance of human resources management in health care: a global context. Hum Resour Health. 2006;4:20. 16872531 10.1186/1478-4491-4-20 PMC1552082

[11] Tursunbayeva A. Human resource management information systems in healthcare. Processes of development, implementation and benefits realization in complex organizations. Milan: Franco Angeli; 2018.

[12] Andersen MK. Human capital analytics: the winding road. J Organization Effective: People Perform. 2017;4(2):133–6.

[13] Sousa MJ, Pesqueira AM, Lemos C, et al. Decision-making based on Big Data Analytics for people management in healthcare organizations. J Med Syst. 2019;43:290. 31332535 10.1007/s10916-019-1419-x

[14] Wang Y, Hajli N. Exploring the path to big data analytics success in healthcare. J Bus Res. 2017;70:287–99.

[15] Bossen C, Bertelsen PS. Digital health care and data work: who are the data professionals? Health Inform Manage J. 2023. 10.1177/18333583231183083 37491822

[16] Menachemi N, Burkhardt J, Shewchuk R, Burke D, Brooks R. Hospital information technology and positive financial performance: a different approach to ROI. J Healthc Manag. 2006;51:1. 16479749

[17] OECD. Health in the 21st Century: putting data to work for stronger health systems. OECD Health Policy studies. Paris: OECD Publishing; 2019.

[18] Tursunbayeva A, Di Lauro S, Pagliari C. People analytics – a scoping review of conceptual boundaries and value propositions. Int J Inf Manag. 2018;43:224–47.

[19] McIver D, Lengnick-Hall ML, Lengnick-Hall CA. A strategic approach to workforce analytics: integrating science and agility. Bus Horiz. 2018;61(3):397–407.

[20] Konovalova VG, Aghashyan RV, Galazova SS. Perspectives and restraining factors of HR analytics in the conditions of digitization of human resources management. In: E.G. Pokova, V.N. Ostrovskaya, V.N., Bogoviz, editors: Socio-economic Systems: Paradigms for the Future. Studies in Systems, Decision and Control book series, Springer, Cham. 2021;314:1015–1024.

[21] Francis H, Keegan A. The changing face of HRM: in search of balance. Hum Resource Manage J. 2006;16:3.

[22] Fernandez V, Gallardo-Gallardo E. Tackling the HR digitalization challenge: key factors and barriers to HR analytics adoption. Competitiveness Review: Int Bus J. 2021;31(1):162–87.

[23] Marler JH, Boudreau JW. An evidence-based review of HR Analytics. Int J Hum Resource Manage. 2017;28:1.

[24] Lawler III, Levenson EE, A., Boudreau J. W. HR metrics and analytics–uses and impacts. Hum Resource Plann J. 2004;27(4):27–35.

[25] Rasmussen T, Ulrich D. Learning from practice: how HR analytics avoids being a management fad. Organ Dyn. 2015;44(3):236–42.

[26] Levenson A, Fink A. Human capital analytics: too much data and analysis, not enough models and business insights. J Organizational Effectiveness: People Perform. 2017;4(2):145–56.

[27] Peeters T, Paauwe J, Van De Voorde K. People analytics effectiveness: developing a framework. J Organizational Effectiveness: People Perform. 2020;7:2:203–19.

[28] Dahlbom P, Siikanen N, Sajasalo P, Jarvenpää M. Big data and HR analytics in the digital era. Baltic J Manage. 2020;15(1):120–38.

[29] KPMG. The future of HR 2019. In the Know or in the No. The Gulf between Action and Inertia. kpmg.com: KPMG International; 2019.

[30] Wirges F, Neyer AK. Towards a process-oriented understanding of HR analytics: implementation and application. RMS. 2023;17:6.

[31] Van den Heuvel S, Bondarouk T. The rise (and fall?) Of HR analytics: a study into the future application, value, structure, and system support. J Organizational Effectiveness: People Perform. 2017;4:2.

[32] Mer A, Virdi AS et al. Navigating the paradigm shift in HRM practices through the lens of artificial intelligence: A post-pandemic perspective. In Tyagi, P., editors, The adoption and effect of artificial intelligence on human resources management, Part A. Emerald Publishing. 2023; 123–154.

[33] Vargas R, Yurova YV, Ruppel CP, Tworoger LC, Greenwood R. Individual adoption of HR analytics: a fine-grained view of the early stages leading to adoption. Int J Hum Resource Manage. 2018;29:22:3046–67.

[34] Bondarouk T, Parry E, Furtmueller E, Electronic. HRM: four decades of research on adoption and consequences. Int J Hum Resource Manage. 2017;28(1):98–131.

[35] Minbaeva DB. Building credible human capital analytics for organizational competitive advantage. Hum Resour Manag. 2018;57:3:701–13.

[36] Angrave D, Charlwood A, Kirkpatrick I, Lawrence M, Stuart M. HR and analytics: why HR is set to fail the big data challenge. Hum Resource Manage J. 2016;26(1):1–11.

[37] McCartney S, Murphy C, Mccarthy J. 21st century HR: a competency model for the emerging role of HR analysts. Personnel Rev. 2021;50:6:1495–513.

[38] Madhavaiah C, Campus K. Are HR professionals ready to adopt HR analytics? A study on analytical skills of HR professionals. J Adv Res Dyn Control Syst. 2018;10(08):303–8.

[39] Hamilton RH, Sodeman WA. The questions we ask: opportunities and challenges for using big data analytics to strategically manage human capital resources. Bus Horiz. 2020;63(1):85–95.

[40] Giermindl LM, Strich F, Christ O, Leicht-Deobald U, Redzepi A. The dark sides of people analytics: reviewing the perils for organisations and employees. Eur J Inform Syst. 2022;31(3):410–35.

[41] Sakka F, Maknouzi E, M. E. H., Sadok H. Human resource management in the era of artificial intelligence: future HR work practices, anticipated skill set, financial and legal implications. Acad Strategic Manage J. 2022;21:1–14.

[42] Cavanagh J, Bartram T, Walker M, Pariona-Cabrera P, Halvorsen B. Health services in Australia and the impact of antiquated rostering practices on medical scientists: a case for HR analytics and evidenced-based human resource management. Personnel Rev. 2024;53(1):18–33.

[43] Pariona-Cabrera P, Bartram T, Cavanagh J, Halvorsen B, Shao B, Yang F. The effects of workplace violence on the job stress of health care workers: buffering effects of wellbeing HRM practices. Int J Hum Resource Manage. 2024;35(9):1654–80.

[44] Skogli E, Jenssen TB, Eide LS, Theie MG. Helsedata–Store verdier på spill. Rapport, Menon-Publikasjon. 2018. (69).

[45] Tursunbayeva A, Bunduchi R, Franco M, Pagliari C. Human resource information systems in health care: a systematic evidence review. J Am Med Inform Assoc. 2017;24:3. 10.1093/jamia/ocw141 PMC5391731 27707821

[46] Agenda Kaupang. Dataforvaltning og -deling i kommunene [Data management and -sharing in the municipalities]. Rapport, 2020.

[47] Røhne M, Ausen D, Grut L. Verktøy for ressursplanlegging i hjemmetjenesten. OPTET – Optimeringsteknologi i hjemmebaserte tjenester [Tool for resource planning in homebased care. OPTET – Optimalization Technology in homebased services]. Report, SINTEF, 2017:00672. 2017.

[48] Shultz JS, André B, Sjøvold E. Managing innovation in eldercare: a glimpse into what and how public organizations are planning to deliver healthcare services for their future elderly. Int J Healthc Manag. 2016;9:3.

[49] Østensen E, Hardiker NR, Hellsesø R. Facilitating the implementation of standardized care plans in municipal healthcare. Computers Inf Nurs. 2022;40:2. 10.1097/CIN.0000000000000798 PMC8820770 34347643

[50] Braitwaite J. Changing how we think about healthcare improvement. BMJ. 2018;361. 10.1136/bmj.k2014 PMC5956926 29773537

[51] Nesse PJ, Erdal OB. Smart digitalization in Nordic cities and municipalities through internet of things. In: Economics and Finance Readings: Selected Papers from Asia-Pacific Conference on Economics & Finance, 2021. 2022 (July): 33–55. Singapore: Springer Nature Singapore.

[52] Danielsen F. Benefits and challenges of digitalization: An expert study on Norwegian public organizations. In DG. O2021: The 22nd annual international conference on digital government research. 2021 (June): 317–326.

[53] OECD and WHO. State of Health in the EU. Norway – Country Health Profile. 2021.

[54] Gautun H, Syse A. Earlier hospital discharge: a challenge for Norwegian municipalities. Nordic J Social Res. 2017;8:1.

[55] Nilsen ER, Olafsen AH, Halvari H, Grov E. Stuck between a rock and a hard place: the work situation for nurses as leaders in municipal health care. J Multidisciplinary Healthc. 2016;9:153–61. 10.2147/JMDH.S100640 PMC4827593 27103816

[56] NOU 2023:4. (2023): Tid for handling. Personellet i en bærekraftig helse- og omsorgstjeneste [Time to act: The personnel in a sustainable healthcare service]. Ministry of Health and Care Services. 2023.

[57] NAV [Norwegian Labour and Welfare Administration]. NAVs bedriftsundersøking 2023: Redusert mangel på arbeidskraft [Norwegian Labour and Welfare Administration’s company survey 2023: reduced lack of labour supply]. NAV-rapport. 2023;2023:5.

[58] Giddens A. The Constitution of Society: outline of the theory of structure. Berkley, CA: University of California Press; 1984.

[59] Orlikowski WJ, Robey D. Information technology and the structuring of organizations. Inform Syst Res. 1991;2:2.

[60] Giddens A. Agency, structure. Central Problems in Social Theory: Action, structure and contradiction in social analysis. 1979: 49–95.

[61] Curtis S, Gesler W, Smith G, Washburn S. Approaches to sampling and case selection in qualitative research: examples in the geography of health. Soc Sci Med. 2000;50(7–8):1001–14. 10714922 10.1016/s0277-9536(99)00350-0

[62] Statistics Norway. Standard for klassifisering av kommuner etter innbyggertall [Standard for the classification of municipalities according to number of inhabitants]. 1998. https://www.ssb.no/klass/klassifikasjoner/115 Accessed 21 Nov 2023.

[63] Braun V, Clarke V. Using thematic analysis in psychology. Qualitative Res Psychol. 2006;3:2:77–101.

[64] Clarke V, Braun V. Teaching thematic analysis: overcoming challenges and developing strategies for effective learning. Psychol. 2013;26(2):120–3.

[65] Lincoln YS, Guba EG. Naturalistic inquiry. Newberry Park. 1985.

[66] Ulrich D, Dulebohn JH. Are we there yet? What’s next for HR? Hum Resource Manage Rev. 2015;25(2):188–204.

[67] Foster J, McLeod J, Nolin J, Greifeneder E. Data work in context: value, risks, and governance. J Association Inform Sci Technol. 2018;69:12.

[68] Comuzzi M, Patel A. How organisations leverage big data: a maturity model. Industrial Manage Data Syst. 2016;116(8):1468–92.

[69] Bach S. HR and new approaches to public sector management: improving HRM capacity. In: Workshop on Global Health Workforce Strategy. 2001. pp. 9–12.

[70] Bach S, Della Rocca G. The management strategies of public service employers in Europe. Industrial Relations J. 2000;31:2.

[71] Dremel C, Wulf J, Herterich MM, Waizmann J-C, Brenner W. How AUDI AG established Big Data Analytics in its digital transformation. MIS Q Exec. 2017;16:2.

[72] Mustafa G, Solli-Sæther H, Bodolica V, Håvold JI, Ilyas A. Digitalization trends and organizational structure: bureaucracy, ambidexterity or post-bureaucracy? Eurasian Bus Rev. 2022;12:4.

[73] Kraus S, Schiavone F, Pluzhnikova A, Invernizzi AC. Digital transformation in healthcare: analysing the current state-of-research. J Bus Res. 2021;123:557–67.

[74] Carbery R, Cross C, editors. Human resource management. London: Bloomsbury Publishing; 2018.

[75] Ward MJ, Marsolo KA, Froehle CM. Applications of business analytics in healthcare. Bus Horiz. 2014;57:571–82. 25429161 10.1016/j.bushor.2014.06.003 PMC4242091

[76] McDermott A, Keating M. Managing professionals: exploring the role of the hospital HR function. J Health Organ Manag. 2011;25:6. 10.1108/14777261111178556 22256665

[77] Robinson S, Williams I, Dickinson H, Freeman T, Rumbold B. Priority-setting and rationing in healthcare: evidence from the English experience. Soc Sci Med. 2012;75:2386–93. 23083894 10.1016/j.socscimed.2012.09.014

[78] Britnell M, Human. Solving the global workforce crisis in healthcare. Oxford: Oxford University Press; 2019.

[79] Correia T, Gomes I, Nunes P, Dussault G. Health workforce monitoring in Portugal: does it support strategic planning and policy-making? Health Policy. 2020;124:3. 10.1016/j.healthpol.2019.12.014 31932075

[80] Alves J, Meneses R. Silos mentality in healthcare services. In: 11th Annual Conference of the EuroMed Academy of Business. 2018.

[81] Spekter & NHO Service og Handel. Morgendagens omsorgsutfordringer – behov for en velferdsmiks [Tomorrow’s challenges in care – in need of a welfare-mix]. Utvalgsrapport. 2019.

[82] Riksrevisjonen [The Office of the Auditor General of Norway]. Dokument 3:5. (2018–2019) Undersøkelse av tilgjengelighet og kvalitet i eldreomsorgen [Document 3:5 (2018–2019) Assessment of accessibility and quality in eldercare]. Riksrevisjonen. 2018.

[83] Mol A. The logic of care: Health and the problem of patient choice. London and New York: Routledge; 2008.

