# Supplementary material for: What influences the use of HR analytics in Human Resource management in Norwegian municipal health care services?
Source: BMC Health Serv Res. 2024 Sep 27;24:1131. doi: 10.1186/s12913-024-11610-y (PMC11429618; doi:10.1186/s12913-024-11610-y)
Supplement: Supplementary file 1 — Supplementary Material 1. [file 12913_2024_11610_MOESM1_ESM.pdf]

Additional file 1: Interview topic guide for the study entitled: "The use of HR analytics in Human Resource Management in Norwegian municipal health care services".

(The original guide was in Norwegian and has been translated into English for documentation purposes).

Formalities: information about the purpose of the study, why they have been requested to participate, that participation is voluntary, and withdrawal is possible at any time, what happens to the result of the study, researcher contact information.

Questions all interviewees:

- Your role/position
- How long you've worked in your current position?
- Earlier carrier

Questions for HR

- Where is HR located in the organization?
- How many works in HR?
- HR's role and tasks?
- HR's function in relation to the municipal administration and management/ the healthcare services?
- General impression of the awareness of HR's competency in the organization?
- Which HR ICT systems are used in HR?
- How integrated are these, compilation of data possible?
- What types of data are collected by HR? (+ what data should be collected?)
- What data or reports from HR are requested by the organisational management team/by the health services? + what are they used for? (similarities/differences apparent across the organization?)
    - Your thoughts about the background for this
- What is your understanding of HR analytics?
- In your understanding, what influences the use/lack of use of data from HR?
- Possible benefits of use of HR data in the municipal organization/health care services?
- Opportunities that lie there for more use of HR data as a basis for decision making.
- Going forward – what happens in terms of changes in relation to HR in the near future? (role, tasks, introduction of ICT-systems, competency)
    - if new systems – what are the expected benefits of adopting such a system?

Questions for chief municipal officers and section managers:

- HR's role and tasks in relation to the municipal administration and management/ the healthcare services?
- What is your understanding of HR analytics?
- What data or reports from HR are requested by the organisational management team/by the health services? + what are they used for? Potential that you see for use of HR data that is not taken advantage of currently?
- General use of digital data for the management and planning of services?
